# 新生淋巴管及其受侵对非小细胞肺癌的预后价值

**DOI:** 10.3779/j.issn.1009-3419.2012.11.09

**Published:** 2012-11-20

**Authors:** 忠吾 胡, 文利 王, 杰 张, 锋 毛, 屠阳 申

**Affiliations:** 1 200030 上海，上海交通大学附属胸科医院/上海市肺部肿瘤临床医学中心胸外科 Department of Thoracic Surgery, Shanghai Chest Hospital/Shanghai Lung Tumor Clinical Medical Center, Shanghai 200030, China; 2 223300 淮安，南京医科大学附属淮安医院胸外科 Department of Thoracic Surgery, Huaian Hospital, Affliated to Nanjing Medical University, Huaian 223300, China; 3 200065 上海，同济大学附属同济医院心胸外科 Department of Cardiothoracic Surgery, Tongji Hospital, Affiated to Tongji University, Shanghai 200065, China; 4 200030 上海，上海交通大学附属胸科医院/上海市肺部肿瘤临床医学中心病理科 Department of Pathology, Shanghai Chest Hospital/Shanghai Lung Tumor Clinical Medical Center, Shanghai 200030, China

**Keywords:** 肺肿瘤, 新生淋巴管, 淋巴管受侵, 预后, Lung neoplasms, Lymphangiogenesis, Lymphatic vessel invasion, Prognosis

## Abstract

**背景与目的:**

已有的研究表明新生淋巴管与多种肿瘤的进展和淋巴转移相关，本研究拟探讨非小细胞肺癌病灶内（旁）新生淋巴管密度及其受肿瘤侵袭状况对非小细胞肺癌的预后价值。

**方法:**

选择特异性单克隆抗体D2-40标记新生淋巴管内皮细胞，以免疫组化方法检测79例Ⅱ期-Ⅲ期非小细胞肺癌病灶内及其中部分病灶旁新生淋巴管表达及其受侵状况，结合患者临床、病理及随访资料，判断其对患者预后的影响和评估价值。

**结果:**

① 病灶内新生淋巴管密度：N2患者高于N0患者（*P*=0.015），新生淋巴管受侵患者明显高于未受侵患者（*P*=0.009），肺癌低分化明显高于高分化肺癌（*P*=0.007），腺癌高于鳞癌（*P*=0.025），患者生存率与之呈负性相关（*P*=0.007）；②病灶旁新生淋巴管密度与预后无明显相关性；③N2肺癌病灶内新生淋巴管受侵多于N0肺癌；④病灶内和病灶旁新生淋巴管受侵患者的生存率低于未受侵患者。

**结论:**

病灶内新生淋巴管密度是影响非小细胞肺癌患者预后的重要因素，可作为判断预后的指标。新生淋巴管受侵的预后意义值得关注。

## Abstract (en)

**Background and objective:**

Studies have shown that tumor metastasis in a variety of tumors is associated with lymphangiogenesis and lymphatic vessel invasion (LVI). Tumor metastasis is an important factor that affects the prognosis of patients. The aim of this study is to determine the prognostic value of lymphangiogenesis and LVI in non-small cell lung cancer (NSCLC).

**Methods:**

We marked the endothelial cells of lymph vessels in lymphangiogenesis with specific monoclonal antibody D2-40. Immunohistochemistry was used to detect the expression of lymphangiogenesis and LVI in 79 cases of stage Ⅰ-Ⅲ NSCLC.

**Results:**

The intratumoral lymphatic vessel density (ITLVD) was significantly higher in patients with N2 disease than those with N0 disease (*P*=0.015). The ITLVD was significantly higher in patients with LVI+ than that in those with LVI- (*P*=0.009). The ITLVD was also remarkably higher in poorly differentiated tumors than that in highly differentiated ones (*P*=0.007). The ITLVD was remarkably higher in adenocarcinoma than that in squamous cell carcinomas (*P*=0.025). *Kaplan-Meier* revealed that the survival rates of patients with higher ITLVD were remarkably poorer than those with lower ITLVD (*P*=0.007). Thus, the ITLVD is an important prognostic factor of NSCLC. The peritumoral lymphatic vessel density is not correlated with the prognosis.

**Conclusion:**

The ITLVD level is an important prognostic factor of NSCLC.

淋巴系统是原发性非小细胞肺癌（以下简称肺癌）主要的转移途径，也是影响患者预后的重要因素^[1, 2]^。随着新型淋巴内皮特异性标志物的开发，新生淋巴管形态学及肿瘤淋巴转移机制的研究进步卓著，在肺癌淋巴转移领域的相关工作尤其活跃。本研究仅着眼于肺癌病灶及其周围组织新生淋巴管的密度和受侵状况，探讨其对肺癌患者预后的判断价值。

## 1 资料与方法

### 1.1 患者资料

#### 1.1.1 纳入标准

2001年1月-2004年12月在上海市胸科医院手术治疗的肺癌患者，术后随访资料完整。符合下列条件者纳入研究：①肺癌完全性切除（参照2005年IASLC肺癌完全性切除手术标准^[3]^）；②术前排除远处转移；③术后病理为鳞癌、腺癌或腺鳞癌；④术后病理分期按2009版UICC肺癌分期标准^[4]^明确为Ⅰ期-Ⅲ期。剔除其中术前行放化疗及术后非肿瘤原因死亡病例。

#### 1.1.2 资料收集

入组患者共79例，其中石蜡组织标本中含病灶旁组织者45例，详见表 1。随访截止日期为2009年6月8日，随访资料均来源于上海市疾病预防控制中心。采集以下资料：住院号、性别、年龄、手术日期、手术类型、病理类型、分化程度、T分期、N分期、淋巴结清扫数量及站别、淋巴结转移情况。

**1:** 入组患者临床特征 The characteristics of eligible patient

Characteristics	Total eligible patients			Peritumoral tissue group	
	Cases (*n*)	Propotion (%)		Cases (*n*)	Propotion (%)
Age (yrs)					
> 60	36	45.6		23	51.1
≤60	43	54.4		22	48.9
Gender					
Male	52	65.8		34	75.6
Female	27	34.2		11	24.4
T stage					
T1a	4	5.1		2	4.4
T1b	30	38.0		19	42.2
T2a	27	34.2		17	37.8
T2b	10	12.6		4	8.9
T3	8	10.1		3	6.7
N stage					
N0	47	59.5		37	82.2
N2	32	40.5		8	17.8
Stage					
Ⅰ	40	50.6		32	71.1
Ⅱ	7	8.9		5	11.1
Ⅲ	32	40.5		8	17.8
Pathological type					
Adenocarcinoma	37	46.8		18	40.0
Squamous	24	30.4		17	37.8
Adenosquamous	18	22.8		10	22.2
Grade of differentiation					
High	52	65.8		33	73.3
Low	27	34.2		12	26.7

### 1.2 研究方法

#### 1.2.1 研究对象

肺癌患者术后病灶石蜡标本，共计79块。

#### 1.2.2 方法与程序

① 将病灶石蜡块切成3 μm厚度，二甲苯脱蜡至水化后，3%H_2_O_2_处理10 min去除过氧化物酶，放入盛有柠檬酸盐缓冲液（pH7.4）的容器中，高压蒸汽修复抗原，煮沸即可，室温冷却，切片再用PBS冲洗3次，每次3 min；②D2-40单克隆抗体（Myboisource, US）采用PBS稀释，按1:50浓度进行稀释，封闭靶抗原后，4 ℃冰箱下恒温储存；③二抗加兔抗鼠Envinsion（DAKO公司，US）室温30 min温育，PBS冲洗3次；④滴加新鲜配制DAB显色液（DAKO公司，US），显微镜下观察5 min-10 min，在显色最佳时用PBS冲洗，中止显色。苏木素复染细胞核，然后水洗、蓝化、脱水、中性树胶封片。

#### 1.2.3 病灶内新生淋巴管密度的定量测定

病灶内新生淋巴管的密度（intratumoral lymphatic vessel density, ITLVD）的评测根据Weidner^[5]^方法。光学显微镜下观察切片，先在100倍镜下选择有明确染色表达的新生淋巴管区域（俗称“热点”区域），每个切片样本选择5个“热点”区域，继而在200倍镜下计数每例切片新生淋巴管数量，单位为个，以Mean±SD表示。

#### 1.2.4 病灶旁新生淋巴管密度的定量测定

病灶旁组织新生淋巴管密度（peritumoral lymphatic vessel density, PTLVD）定义为在肿瘤边缘附近500 μm区域内的新生淋巴管密度。由于多数的标本癌旁组织较少，故在镜下取2个-5个“热点”区域，200倍镜下观察计数每例切片单位视野平均新生淋巴管数，单位为个，以Mean±SD表示。

#### 1.2.5 新生淋巴管受侵的定义

即新生淋巴管受到肿瘤细胞侵犯（lymphatic vessels invasion, LVI），镜下病灶中或病灶旁组织中的新生淋巴管管腔中存在肿瘤细胞团，即为新生淋巴管受侵，以LVI+表示，无新生淋巴管受侵以LVI-表示。

### 1.3 统计学分析

所有数据均采用SPSS 17.0统计软件包进行处理。分析总体率或构成比之间有无差异采用χ^2^检验。生存分析采用*Kaplan-Meier*乘积法和*Log-rank*检验，多因素分析采用*Cox*回归模型。由于新生淋巴管密度呈非正态分布，相关因素比较采用*Mann-Whitny U*检验。以*P* < 0.05为差异有统计学意义。

## 2 结果

### 2.1 病灶内新生淋巴管及其受侵

#### 2.1.1 病灶内新生淋巴管的表达及其形态

D2-40特异性标记的新生淋巴管显示为棕黄色的单层薄壁管腔，管壁无肌细胞和周细胞，管腔内无红细胞。病灶内新生淋巴管的典型图像见图 1及其描述。

**1:**
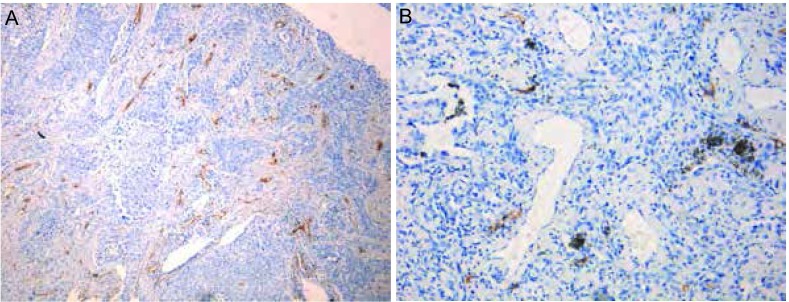
病灶内新生淋巴管的表达。A：40倍显微镜下所显示的鳞癌组织内新生淋巴管的表达；B：100倍显微镜下所显示的腺癌组织内新生淋巴管的表达。 The expression of lymphangiogenesis in tumor tissue under microscope. A: The expression of lymphangiogenesis in lung squamous carcinoma tissue (×40, IHC stain, brown granules); B: The expression of lymphangiogenesis in lung adenocarcinoma tissue (×100, IHC stain, brown granules).

#### 2.1.2 ITLVD

ITLVD为11.3±7.9。对相关因素进行*Mann-Whitny U*检验，研究病灶内新生淋巴管密度与临床病理特征的关系，结果详见表 2。

**2:** 病灶内新生淋巴管密度/淋巴管受侵与临床病理特征的关系 The relationship between ITLVD/LVI and clinicopathological characteristics

Characteristics	ITLVD (Mean±SD)	*P*	LVI (-/+)	*P*
Gender		0.385		0.554
Male	12.6±8.5		18/19	
Female	10.7±7.6		38/14	
Age (yrs)		0.605		0.798
> 60	10.8±7.5		25/11	
≤60	12.0±8.5		31/12	
LVI		0.009		
(+)	15.3±9.2			
(-)	9.7±6.8			
Tumor size (cm)		0.436		0.094
≤3	9.8±4.9		28/6	
> 3-≤5	11.6±10.7		16/12	
> 5	13.1±8.8		12/5	
N stage		0.015		0.019
N0	9.2±5.4		38/9	
N2	14.5±9.9		18/14	
Grade of differentiation		0.007		0.101
High	9.1±6.5		16/11	
Low	15.6±8.7		40/12	
Pathological type		0.025		0.179
Adenocarcinoma	13.1±7.8		20/4	
Squamous	7.8±6.1		26/11	
Adenosquamous	12.4±9.1		10/8	
ITLVD: intratumoral lymphatic vessel density; LVI: lymphatic vessel invasion.				

#### 2.1.3 病灶内新生淋巴管受侵

47例N0组患者中有9例LVI+，而32例N2组患者中有14例LVI+，两组间存在统计学差异（*P*=0.019），病灶内新生淋巴管受侵的典型图像见图 2及其描述，而性别、年龄、肿瘤直径、肿瘤分化程度和病理类型等因素与之无相关性，具体数据详见表 2。

**2:**
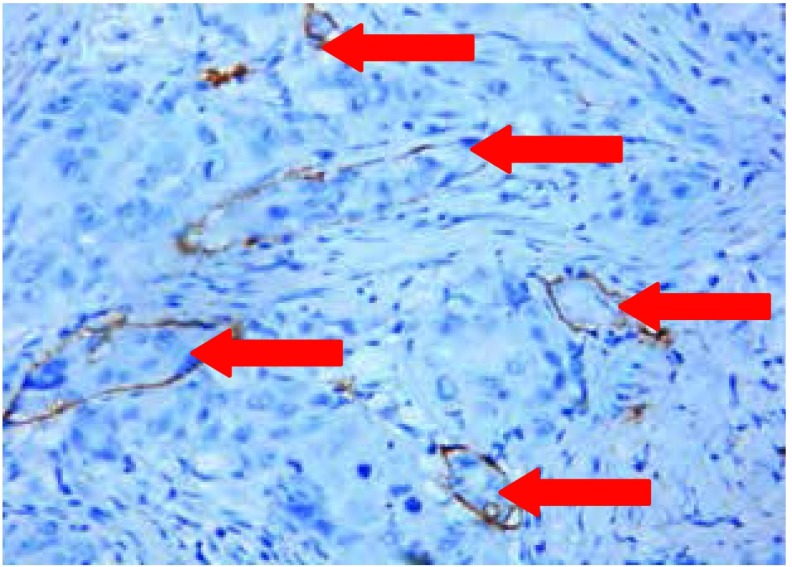
病灶内新生淋巴管受侵 The red arrow showed tumor cell invade the lymphangiogenesis in tumor tissue (Squamous carcinoma, IHC stain, original magnification×200).

#### 2.1.4 生存分析

79例患者中，低ITLVD组者（≤11.3个）35例，高ITLVD组者（> 11.3个）44例，其总体5年生存率为57%。单因素生存分析显示高ITLVD（*P*=0.007）及LVI+（*P*=0.004）是影响预后的重要因素（图 3）。*Cox*多因素回归模型显示：高ITLVD、纵隔淋巴结转移、肿瘤直径及分化程度是影响患者预后的重要因素（表 3）。

**3:**
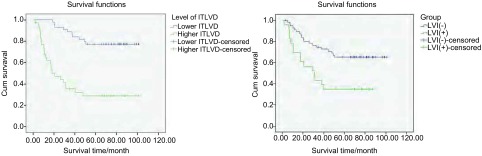
*Kaplan-Meier*累计生存时间曲线分析。A：高ITLVD组患者和低ITLVD组患者的*Kaplan-Meier*累计生存时间曲线；B：病灶LVI+组与LVI-组的*Kaplan-Meier*累计生存时间曲线。 *Kaplan-Meier* cumulative survival time curves analysis. A: *Kaplan-Meier* cumulative survival time curves of higher ITLVD (> 11.3) and lower ITLVD (≤11.3); B: *Kaplan-Meier* cumulative survival time curves of LVI+ group and LVI- group in tumor tissue.

**3:** 79例非小细胞肺癌患者生存期的预后因素（*Cox*回归分析） Prognostic factors for survival in 79 non-small cell lung cancer (NSCLC) patients (*Cox* regression model)

Characteristics	*P*	95%CI
Level of ITLVD	0.001	1.789-9.230
Tumor size	0.038	1.028-2.611
Grade of differentiation	0.017	0.171-0.844
N stage	0.003	2.059-12.318

### 2.2 病灶旁新生淋巴管及其受侵

#### 2.2.1 病灶旁新生淋巴管的表达及典型图像

79例入组患者中，含病灶旁组织者共45例，病理特征详见表 1。病灶旁新生淋巴和病灶内新生淋巴管形态相似，非均匀出现在病灶旁组织中。其特征性典型图像见图 4及其描述。

**4:**
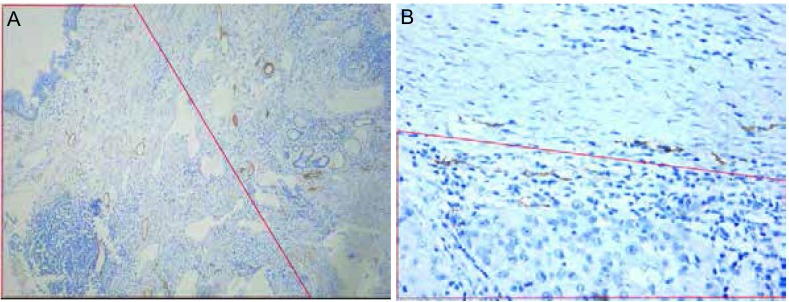
病灶旁新生淋巴管的表达（红色梯形区域）。A：100倍显微镜下所显示的腺癌癌旁组织内新生淋巴管的表达；B：200倍显微镜下所显示的腺鳞癌癌旁组织内新生淋巴管的表达。 The red trapezoidal area showed the expression of lymphangiogenesis in peritumor tissue under microscope. A: Lung adenocarcinoma (×100, IHC stain, brown granules); B: Lung adenosquamous carcinoma (×200, IHC stain, brown granules).

#### 2.2.2 PTLVD

45例患者PTLVD为（7.8±5.2）个。对相关因素进行*Mann-Whitny U*检验，结果显示：病灶旁组织新生淋巴管的表达与患者性别、年龄、LVI、肿瘤直径、淋巴结分期、肿瘤分化程度及病理类型等无相关性（表 4）。

**4:** 病灶旁新生淋巴管密度/淋巴管受侵与临床病理特征的关系 The relationship between PTLVD/LVI and clinicopathological characteristics

Characteristics	PTLVD (Mean±SD)	*P*	LVI (-/+)	*P*
Gender		0.137		0.570
Male	6.9±4.2		26/8	
Female	10.5±7.1		7/4	
Age (yrs)		0.856		0.728
> 60	8.0±5.4		17/6	
≤60	7.6±5.1		16/6	
LVI		0.534		
(+)	8.0±4.6			
(-)	7.7±5.5			
Tumor size (cm)		0.381		0.209
≤3	7.5±4.7		18/3	
> 3-≤5	7.8±5.2		10/7	
> 5	9.2±6.3		5/2	
N stage		0.19		0.119
N0	7.5±5.2		29/8	
N2	9.3±5.5		4/4	
Grade of differentiation		0.665		0.103
High	8.1±5.6		25/8	
Low	6.9±3.8		8/4	
Pathological type		0.072		0.185
Adenocarcinoma	8.7±5.4		14/3	
Squamous	5.7±3.6		13/5	
Adenosquamous	9.9±6.3		6/4	
PTLVD: peritumoral lymphatic vessel density.				

#### 2.2.3 病灶旁新生淋巴受侵

45例患者中LVI+者共12例。LVI+与患者性别、年龄、肿瘤直径、淋巴结分期、肿瘤分化程度及病理类型等亦无相关性（表 4）。

#### 2.2.4 生存分析

45例患者分为低PTLVD组（≤7.8个）28例，高PTLVD组（> 7.8个）17例。单因素生存分析显示PTLVD的高低与患者预后无明显相关性（*P*=0.278）；而LVI+仍是影响预后的重要因素（*P*=0.004）（图 5）。*Cox*多因素回归模型显示：淋巴结分期、肿瘤直径及肿瘤分化程度是影响患者术后预后的重要因素（表 5），而与PTLVD水平无明显相关性（*P*=0.704）。

**5:**
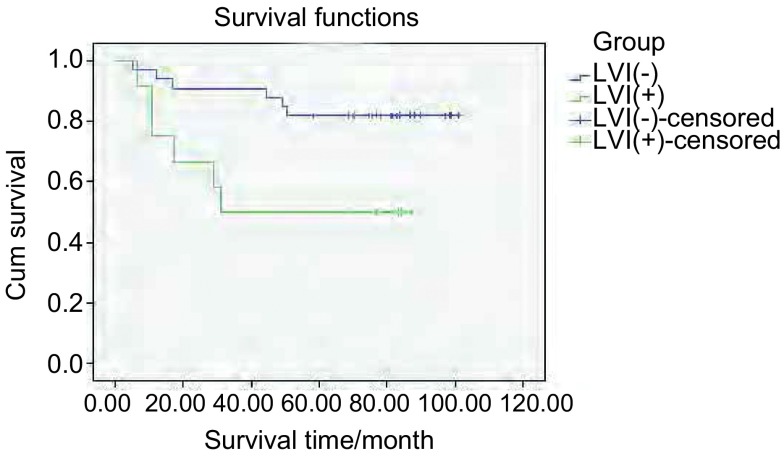
病灶旁LVI+组与LVI-组的*Kaplan-Meier*累计生存时间曲线。 *Kaplan-Meier* cumulative survival time curves of LVI+ group and LVI- group in peritumor tissue.

**5:** 45例NSCLC患者生存期的预后因素（*Cox*回归分析） Prognostic factors for survival in 45 NSCLC patients (*Cox* regression model)

Characteristics	*P*	95%CI
Level of PTLVD	0.704	1.509-25.270
Tumor size	0.002	1.634-9.420
Grade of differentiation	0.043	0.070-1.000
N stage	0.040	1.061-13.872

## 3 讨论

现有研究认为，肿瘤病灶内新生淋巴管是淋巴转移的预设途径，细胞因子则在淋巴管和肿瘤细胞的相互关联中发挥重要作用。原发肿瘤分泌的淋巴生长因子诱导肿瘤病灶及其周围的淋巴管生成，从而构成转移通路。肿瘤细胞则通过这些淋巴管扩散进入淋巴结，并进一步介导远处转移^[6-8]^。随着淋巴内皮特异性标志物的逐渐发现，有关新生淋巴管的研究在机制、形态、功能等层面均有所进展^[9]^。Nakamura等^[10]^对于早期胃癌的研究显示，无论病灶内还是病灶旁新生淋巴管对肿瘤的淋巴转移都有重要意义，是淋巴转移的危险因素之一。Maula等^[11]^对头颈肿瘤的研究发现，病灶内新生淋巴管的表达水平与局部淋巴转移呈正相关，是重要的预后因素。在胰腺肿瘤中亦发现高病灶内淋巴管密度与淋巴及血管侵犯相关，提示病灶新生淋巴管促进肿瘤的恶性进展^[12]^。

本研究发现，高ITLVD与患者纵隔淋巴结转移密切相关，是重要预后因素。提示肿瘤内新生淋巴管可能增加肿瘤细胞与淋巴管的接触面积，促进肿瘤细胞扩散。非小细胞肺癌患者ITLVD和淋巴管侵犯的程度具有相关性，ITLVD越高，癌细胞侵犯淋巴管的可能性越大，越有利于癌细胞的淋巴转移。低分化肿瘤患者的ITLVD也较高，更易发生淋巴转移。这与上述文献报告结论基本相同。肺腺癌患者ITLVD较高，该类患者较之鳞癌更易出现纵隔淋巴结和远处转移。我们前期的工作^[13]^显示，腺癌病灶新生淋巴管密度平均为6.20±3.81，明显高于鳞癌组（3.18±3.95, *P*=0.001），且与患者年龄、性别、T分期及肿瘤分化程度无明显相关性。但亦有研究显示鳞癌病灶中ITLVD较高^[14]^。

在病灶内，本研究单因素分析显示LVI与ITLVD存在正相关性，即病灶中新生淋巴管密度愈高，其受侵的机率亦随之增加。LVI尤其与纵隔淋巴结转移关系较为密切，N0组：9/47者LVI+，N2组：14/32者LVI+，两组统计学差异明显（*P*=0.019）。但在多因素分析中并未出现统计学差异，故尚待进一步扩大样本深入研究。然而值得注意的是，生存率分析提示，无论病灶内还是病灶旁组织，LVI+组与LVI-组的生存曲线较早即出现明显分离，LVI+组生存率急剧下降（图 3B及图 5），提示LVI+对生存率的影响不容小觑，值得进一步研究。

2009年世界肺癌大会发布的第7版肺癌分期，按肿瘤直径细化T分期，更提升了肿瘤直径对预后影响的重要性。本研究中多因素分析亦显示肿瘤直径是影响非小细胞肺癌患者预后的重要因素。Kadota等^[14]^在针对非小细胞肺癌患者的新生淋巴管研究中指出，直径3 cm以上的肿瘤，其新生淋巴管密度明显大于直径3cm以下者。本文表 2亦可发现，直径 < 3 cm的肿瘤ITLVD为（9.8±4.9）个，总体低于肿瘤直径 > 3 cm-≤5 cm和 > 5 cm者（分别为11.6±10.7和13.1±8.8），随着病灶直径的增加ITLVD呈现逐步升高趋势。

本研究通过对45例患者病灶旁组织的分析显示，PTLVD与患者预后无明显相关性（*P*=0.278）。但Sun等^[15]^研究认为，在非小细胞肺癌的转移过程中，病灶旁新生淋巴管发挥着重要作用，而病灶内新生淋巴管可能并无重要意义。结果的不一致可能因该组患者样本量较少，且部分癌旁组织在整张切片中所占面积较小，从而影响对PTLVD的客观评价，故有待扩大样本研究。亦有部分研究^[16, 17]^指出高PTLVD组有较好的预后：在某些肿瘤中，病灶周围数目众多的功能性淋巴管网能够诱发T细胞介导的免疫反应，同时肿瘤细胞通过分泌VEGF-C/D促进新生淋巴管的生成，提高了癌旁组织淋巴管的密度，有利于免疫细胞趋化到肿瘤周围组织，诱导抗肿瘤免疫应答。此外，尽管大部分研究都提示肿瘤的淋巴转移与新生淋巴管有关，但亦有研究指出肿瘤细胞的转移扩散可能是通过预先存在的淋巴管。Leu等^[18]^通过对鼠肉瘤淋巴管的研究，质疑新生淋巴管对肿瘤侵犯及转移的作用，提出在病灶中或者病灶周围原先存在的淋巴管才是影响肿瘤转移的重要因素。这些研究中的结论差异，一方面说明新生淋巴管功能的复杂多样，另一方面也反映了各研究的局限性，有待时日和深入研究，才能全面客观地揭示其真相。

综上所述，虽然存在分歧，但是大多数研究仍然肯定新生淋巴管在肿瘤淋巴转移机制中的重要作用。就我们有限病例的研究而言，病灶内新生淋巴管密度是影响非小细胞肺癌患者预后的重要因素，可作为判断预后的指标。病灶旁新生淋巴管密度的预后意义有待大样本进一步研究。而病灶内和病灶旁新生淋巴管受侵与淋巴转移和生存率关系密切，值得关注。
